# Draft Genome Sequence of *Geobacillus icigianus* Strain G1w1^T^ Isolated from Hot Springs in the Valley of Geysers, Kamchatka (Russian Federation)

**DOI:** 10.1128/genomeA.01098-14

**Published:** 2014-10-23

**Authors:** Alla V. Bryanskaya, Aleksey S. Rozanov, Maria D. Logacheva, Anastasia V. Kotenko, Sergey E. Peltek

**Affiliations:** a Institute of Cytology and Genetics, Siberian Branch of the Russian Academy of Sciences, Novosibirsk, Russian Federation; b Lomonosov Moscow State University, Moscow, Russian Federation

## Abstract

The *Geobacillus icigianus* G1w1^T^ strain was isolated from sludge samples of unnamed vaporing hydrothermal (97°С) outlets situated in a geyser in the Troinoy region (Valley of Geysers, Kronotsky Nature Reserve, Kamchatka, Russian Federation; 54°25′51.40″N, 160°7′41.40″E). The sequenced and annotated genome is 3,457,810 bp and encodes 3,342 genes.

## GENOME ANNOUNCEMENT

In 2001, T. Nazina et al. described the genus *Geobacillus* and transferred six existing *Bacillus* species into it (*Geobacillus stearothermophilus*, *Geobacillus thermocatenulatus*, *Geobacillus thermoleovorans*, *Geobacillus kaustophilus*, *Geobacillus thermoglucosidasius*, and *Geobacillus thermodenitrificans*). In this work, they also proposed two new species for the genus: *Geobacillus subterraneus* and *Geobacillus uzunensis* (1). Afterward, other new species were added to the genus *Geobacillus*: *Geobacillus toebii* (2), *Geobacillus debilis* (3), *Geobacillus lithuanicus* (4), and *Geobacillus gargensis* (5). The phylogeny of other several species is still uncertain (6).

The strain G1w1^T^ was isolated from sludge samples of unnamed vaporing hydrothermal (97°С) outlets situated in the geyser Troinoy region (Valley of Geysers, Kronotsky Nature Reserve, Kamchatka, Russian Federation) (54°25′51.40″N, 160°7′41.40″E). The microbial community was clearly visible without a microscope as a deep-green encrustation on pink sludge sediment.

*G. icigianus* G1w1^T^ culture was cultivated in liquid medium containing 1% trypton and 0.5% yeast extract, and 8 ml of cell culture were pelleted by centrifugation and resuspended in 75 µL of H_2_O by intense pipetting. DNA preparations for genome sequencing were made using the GeneJET DNA purification kit (Fermentas) according to the manufacturer’s instructions. The Nextera DNA Sample Prep kit (Illumina) was used to create libraries for genome sequencing. Genomic DNA was sequenced using the MiSeq Reagent kit version 2 (Illumina) in the Laboratory of Evolutionary Genomics of the Faculty of Bioengineering and Bioinformatics, Moscow State University.

*De novo* assembly of short reads into contigs was performed using SPAdes version 3.1.0. Contigs shorter than 1,000 bp were deleted. A total of 207 contigs yielded a genome sequence 3,457,810 bp in length, and the G+C content is 52%. Open-reading frame prediction and automatic annotation was performed using NCBI PGAAP (http://www.ncbi.nlm.nih.gov/genome/annotation_prok). The draft genome sequence contained 3,342 genes, 3,146 coding DNA sequences, 25 rRNAs (5S, 16S, 23S), 85 tRNAs, and 1 ncRNA.

Phylogenetic analysis was performed using 16S rRNA sequences with the UPGMA algorithm implemented in MEGA version 6, and 16S rRNA sequences of *Geobacillus* type strains were found using the StrainInfo (http://www.straininfo.net) and GenBank (http://www.ncbi.nlm.nih.gov/nucleotide) databases. According to phylogenetic analysis, the strain G1w1^T^ can be assigned to a new species.

### Nucleotide sequence accession numbers.

The draft genome sequence for *G. icigianus* G1w1^T^ has been deposited in DDBJ/EMBL/Genbank under the accession number JPYA00000000. The 207 contigs have been deposited under accession numbers JPYA01000001 to JPYA01000207.
